# Prevalence and type of drug–drug interactions involving ART in patients attending a specialist HIV outpatient clinic in Kampala, Uganda

**DOI:** 10.1093/jac/dkv259

**Published:** 2015-08-18

**Authors:** K. Seden, C. Merry, R. Hewson, M. Siccardi, M. Lamorde, P. Byakika-Kibwika, E. Laker, R. Parkes-Ratanshi, D. J. Back, S. H. Khoo

**Affiliations:** 1 Department of Molecular and Clinical Pharmacology, Institute of Translational Medicine, University of Liverpool, Liverpool, UK; 2 Infectious Diseases Institute, Makerere University College of Health Sciences, Kampala, Uganda; 3 Department of Pharmacology and Therapeutics, Trinity College Dublin, Dublin 2, Ireland; 4 Royal Liverpool and Broadgreen University Hospital NHS Trust, Liverpool, UK

## Abstract

**Objectives:**

Scale-up of HIV services in sub-Saharan Africa has rapidly increased, necessitating evaluation of medication safety in these settings. Drug–drug interactions (DDIs) involving antiretrovirals (ARVs) in sub-Saharan Africa are poorly characterized. We evaluated the prevalence and type of ARV DDIs in Ugandan outpatients and identified the patients most at risk.

**Methods:**

A total of 2000 consecutive patients receiving ARVs at the Infectious Diseases Institute, Kampala were studied. The most recent prescription for each patient was screened for clinically significant DDIs using www.hiv-druginteractions.org. Univariable and multivariable logistic regression were used to identify risk factors for DDIs. A screening tool was developed using significant risk factors and tested in a further 500 patients.

**Results:**

Clinically significant DDIs were observed in 374 (18.7%) patients, with a total of 514 DDIs observed. Only 0.2% of DDIs involved a contraindicated combination. Comedications commonly associated with DDIs were antibiotics (4.8% of 2000 patients), anthelmintics (2.2%) and antifungals (3.5%). Patient age, gender, CD4 count and weight did not affect risk of DDIs. In multivariable analysis, the patient factors that independently increased risk of DDIs were two or more comedications (*P* < 0.0001), a PI-containing ARV regimen (*P* < 0.0001), use of an anti-infective (*P* < 0.0001) and WHO clinical stage 3–4 (*P* = 0.04). A scoring system based on having at least two of these risk factors identified between 75% and 90% of DDIs in a validation cohort.

**Conclusions:**

Significant ARV DDIs occur at similar rates in resource-limited settings and developed countries; however, the comedications frequently causing DDIs differ. Development of tools that are relevant to particular settings should be a priority to assist with prevention and management of DDIs.

## Introduction

Successful scale-up of HIV services in countries such as Uganda may have contributed to an overall increase in functional health facilities, a rise in patient engagement and retention in care.^1^ As more patients access medical care, including ART, evaluating medication safety is increasingly important in these settings.^2^ Current ART is complex due to lifelong treatment comprising multidrug regimens with significant propensity for drug–drug interactions (DDIs), which may result in raising or lowering the concentration of coprescribed drugs or antiretrovirals (ARVs). Elevated drug concentrations may be associated with drug toxicity and lower concentrations may be associated with therapeutic failure. Subtherapeutic drug concentrations are of particular concern when treating HIV, due to the possible emergence of drug-resistant strains, which can compromise the utility of ARVs and reduce future treatment options on an individual patient or population basis. Studies from Europe, the USA and South America have reported a prevalence of clinically significant DDIs of 14%–58%,^3–8^ suggesting DDIs involving ARVs are common, frequently unavoidable but manageable in the majority of cases. However, physician recognition of ARV DDIs has been reported to be low in a UK study.^3^ Few data are available in low-resource settings; the prevalence of DDIs ranged from 14.8%–33.5% in three studies undertaken in Nigeria, Kenya and South Africa.^9–11^

Resource-limited settings in sub-Saharan Africa are likely to face specific risk factors for DDIs. Here, a public health approach is deployed to maximize health gains for the population who require treatment (with most individuals managed using a combination of clinical monitoring and symptom-driven laboratory monitoring), in contrast to individualized therapy in developed countries. Medication recording in clinical notes may be incomplete, as reported in UK studies.^7,12^ This may pose a risk in African settings, where ‘vertical’ (unintegrated) programmes for HIV, TB, malaria and other conditions exist. Detection of subtherapeutic ARV levels is complex in the absence of therapeutic drug monitoring as there may be a delay between the index prescribing event and the emergence of clinical or virological failure. In the developed world, management of DDIs may involve substitution of a medication or adjustment of doses. Laboratory monitoring is routinely utilized in such situations, but is largely unavailable in resource-limited settings. It is therefore vital that the common interactions of clinical importance in such settings are recognized and detected, in order to prevent patient harm.

We studied the prevalence and type of DDIs involving ARVs in a large Ugandan outpatient ARV programme in order to identify factors associated with risk of having DDIs and to develop a simple screening tool to identify patients most at risk of having clinically significant DDIs in this setting.

## Patients and methods

### Setting

The Infectious Diseases Institute (IDI) in Kampala, Uganda, is the national referral centre for HIV treatment and, at the time of the study, was providing care for >9000 patients, of whom 6832 were taking WHO standard ARV regimens during May 2012. Unusually for sub-Saharan Africa, patient records and prescribing are via an electronic system, the Integrated Care Enterprise Application (ICEA), which integrates electronic health records with electronic prescribing and dispensing, and pathology and radiology reporting.

### Ethical considerations

This is a retrospective study on a large dataset, involving no intervention or patient contact. Local confidentiality and data protection agreements were adhered to throughout the study. The protocol for the retrospective use of data routinely collected at IDI was reviewed and approved by the Makerere University Faculty of Medicine Research and Ethics Committee (approval number: 120-2009) and the Uganda National Council for Science and Technology (approval number: HS 683). According to the protocol procedures, patients do not provide verbal or written consent, but all their information is analysed after stripping it of unique personal identifiers. Linked anonymized data were subsequently used by the clinical service team to trace all patients with contraindicated drug combinations.

### Data collection

A sample of 2000 consecutive patients taking current ARVs and accessing care at IDI was selected from the clinic database. Exploratory analysis at the halfway point (1000 patients)^13^ was equivalent to analysis of the 2000 patient sample. The final analysis was therefore concluded at 2000 patients. This sample represents a third of the adult clinic population who were receiving ART at the time of the study. Patient demographics and current medication were recorded in an anonymized database. As patients were not approached to give a full medication history, only medicines prescribed from IDI were recorded.

The most recent prescription for each patient was screened for DDIs using www.hiv-druginteractions.org. This is a comprehensive database containing >11 000 HIV DDIs, which is widely utilized throughout Europe and since 2012 has included all drugs from the WHO Model List of Essential Medicines. The clinical significance of DDIs was assessed and corroborated by three of the authors (K. S., R. H. and S. K.) using a previously developed technique evaluating the likelihood of interaction, the therapeutic index of the affected drug(s) and the severity of the potential adverse effect.^3,6^ A quality-of-evidence rating system has been developed and applied to all interactions in the database, in order to aid clinical decision making. Potential DDIs excluded from the analysis were those between the ARV regimen such as tenofovir and lopinavir and between ARVs and co-trimoxazole. Such combinations are widely used in all settings, with low likelihood of adverse effects of clinical significance. Co-trimoxazole was, however, included as a comedication in the analysis. Interactions between ARVs that were considered to be clinically relevant, e.g. between efavirenz and lopinavir/ritonavir, were included. Fixed-dose combinations, e.g. artemether/lumefantrine-containing antimalarials, were considered in the analysis as one comedication.

### Statistical analyses

Relationships between patient characteristics and DDI risk were evaluated. In descriptive analysis, a *t*-test was used for means of continuous variables (Mann–Whitney *U*-test for non-normally distributed variables: CD4 count) and a *χ*^2^ test was used for categorical variables. The ‘cut-offs’ of interest for continuous variables such as number of comedications were determined visually. Univariable analysis was used to determine the patient factors and medication that conferred higher risk of DDIs. Multivariable logistic regression was used to determine whether the following independent variables increased patient risk of DDIs involving ARVs: age, gender, CD4 count, patient weight, WHO clinical stage, number of comedications, type of comedications and ARV regimen. Forward stepwise selection was used to fit the model.

A risk factor scoring system was developed using cross-tabulation of the independent variables found to confer higher risk of DDIs. To use this system, one ‘point’ was assigned for each risk factor possessed by the patient and summed together to give a score. Sensitivity and specificity analysis (ROC curve) was undertaken in a test set of 500 consecutive patients from the same clinic cohort, which were not included in the main dataset [prevalence of DDIs: 97 (19.4%); contraindicated combinations: 2 (0.4%)]. In order to identify the most efficient methods to use in this low-resource setting, scoring system models using different combinations of significant risk factors were compared for more favourable sensitivity and specificity and ease of use.

Analyses were carried out using the SPSS statistical package (v.21; IBM, NY, USA) and STATA (v.13; STATA, TX, USA).

## Results

### Patient characteristics

Patient characteristics are shown in Table 1. Patients were predominantly female (65.2%) and the average age was 40.4 years. The median CD4 count was 391 cells/mm^3^, with 266 (13.3%) patients taking a second-line (PI-containing) ARV regimen. Almost all (99.7%) patients were taking one or more comedication alongside their ARV regimen, with a mean of 1.9 comedications per patient recorded.

**Table 1.** Prevalence of drug interactions for different patient characteristics

Patient factor	All	At least one DDI	No DDIs	*P*^a^
All	2000	374 (18.7%)	1626 (81.3%)	—
Age (years), mean (SD)	40.4 (9.05)	40.2 (8.54)	40.4 (9.16)	0.742
Gender	female: 1305 (65.2%)	230 (17.6%)	1075 (82.4%)	0.091
	male: 695 (34.8%)	144 (20.7%)	551 (79.3%)	
Weight (kg), mean (SD)	62.0 (11.48)	61.1 (10.83)	62.2 (11.61)	0.080
CD4 count (cells/mm^3^), median (range)	391 (4–2603)	369 (4–1666)	397 (5–2603)	0.066
Second-line (PI-containing) regimen	266 (13.3%)	82 (30.8%)	184 (69.2%)	<0.0001
Comedications				<0.0001
0	6 (0.3%)	2 (33.3%)^b^	4 (66.7%)	
1	897 (44.9%)	34 (3.8%)	863 (96.2%)	
2	406 (20.3%)	94 (23.2%)	312 (76.8%)	
3	663 (33.2%)	231 (34.8%)	432 (65.2%)	
≥4	28 (1.4%)	13 (46.4%)	15 (53.6%)	
WHO stage				<0.0001
1	89/1997 (4.5%)	9 (10.1%)	80 (89.9%)	
2	483/1997 (24.2%)	73 (15.1%)	410 (84.9%)	
3	763/1997 (38.2%)	131 (17.2%)	632 (82.8%)	
4	662/1997 (33.1%)	161 (24.3%)	501 (75.7%)	

^a^*t-*test/Mann–Whitney *U*-test used for means of continuous variables and χ^2^ test used for categorical variables.

^b^Interactions between ARVs only.

### Prevalence and type of drug interactions

A total of 374 (18.7%) patients had one or more clinically significant DDI, with 514 clinically significant interactions identified in total. Comedication use and prevalence of DDIs with each class of comedication is shown in Figure S1 (available as Supplementary data at *JAC* Online). Comedications commonly associated with potential DDIs were antibiotics [103 (20.0%) of 514 interactions, *P* < 0.0001], antifungals [87 (16.9%), *P* < 0.0001] and anthelmintics [81 (15.8%), *P* < 0.0001]. These affected 95 (4.8%), 70 (3.5%) and 43 (2.2%) patients, respectively. Antimalarial, steroid and sedative/anxiolytic use was also significantly associated with DDIs (*P* < 0.0001); however, patient numbers were low in this cohort. Contraindicated drug combinations were observed in four patients (0.2%), all involving nevirapine and ketoconazole.

The observed interactions resulted from the following mechanisms: modulation of/competition for metabolic pathways, 359 (69.8%); modulation of/competition for renal elimination of unchanged drug, 40 (7.8%); and overlapping toxicity, 115 (22.4%).

### Risk factor analysis

Table 1 shows the prevalence of drug interactions by patient characteristic. From this descriptive analysis, the cut-offs of at least two comedications and WHO stage 3–4 were considered relevant. The final multivariable logistic regression model was fitted by forward stepwise selection and included the use of two or more comedications, a PI-containing regimen, use of an anti-infective (antibiotic, antifungal, antimalarial or anthelmintic) and WHO clinical stage 3–4 (Table 2). These four factors independently increased the risk of DDIs.

**Table 2.** Logistic regression analysis of patient factors contributing to risk of DDIs

Multivariable logistic regression analysis^a^		
variable	OR (95% CI)	*P*
At least two comedications	3.4 (2.3–5.1)	<0.0001
Second-line (PI-containing) regimen	2.8 (1.9–4.1)	<0.0001
WHO stage 3–4	1.4 (1.0–1.9)	0.04
Anti-infective	11.5 (8.4–15.7)	<0.0001

^a^Variables removed by forward stepwise regression: weight, CD4 count and gender.

**Figure 1.**
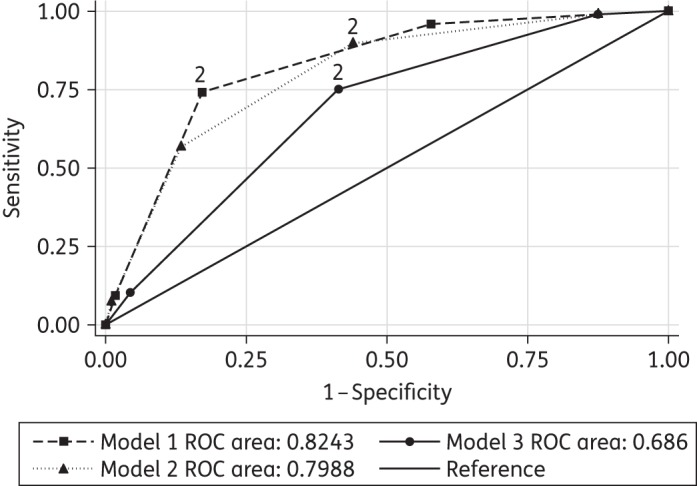
ROC curve analysis showing sensitivity and specificity of the screening tool models for detecting patients with DDIs. The point for each model which relates to two risk factors, the chosen cut-off, is indicated.

A risk factor scoring tool was developed incorporating the four patient factors that were found in descriptive analysis and logistic regression to significantly increase risk of DDIs. A risk score value of 2 (i.e. two or more risk factors out of four) was selected as the cut-off value for screening, based on ROC curve sensitivity and specificity. A model based on four risk factors was sensitive; however, specificity was low, requiring the screening of 52.8% of the cohort to detect 89.7% of the patients with DDIs. This was then compared with risk score models using combinations of only three of these factors (Figure 1).

The overall ability of the risk score models to differentiate those with DDIs from those without ranged from 0.69–0.82 (AUC; see Figure 1 and Table 3).

**Table 3.** Performance of screening tool models for detecting patients with DDIs using combinations of risk factors

Risk score: Screen patients with two or more of the following risk factors	ROC AUC (95% CI)	Sensitivity	Specificity	Patients screened (%)	Patients with DDIs detected (%)	Number of DDIs detected (% of DDIs in cohort)	Contraindicated combinations detected (test sample, 2/500; main sample, 4/2000)
Model 1	0.80 (0.76–0.83)	89.7%	56.1%	264 (52.8)	87 (89.7)	133 (91.1)	T: 2
WHO stage 3–4							M: 4
anti-infective							
PI regimen							
at least two comedications							
Model 2	0.82 (0.79–0.86)	74.2%	82.9%	141 (28.5)	72 (74.2)	109 (74.7)	T: 2
anti-infective							M: 4
PI regimen							
at least two comedications							
Model 3	0.69 (0.64–0.73)	75.2%	58.5%	240 (48.0)	73 (75.3)	114 (78.0)	T: 2
WHO stage 3–4							M: 3
PI regimen							
at least two comedications							

T, test sample; M, main sample.

Table 3 shows the number and proportion of patients from the 500 patient test dataset that would have been screened using each model and the number of patients with DDIs and total number of DDIs detected, if the tool was used to prioritize high-risk patients for DDI screening.

## Discussion

Patients in this African outpatient setting were found to take a range of prescribed comedications. One or more clinically significant DDI affected 18.7% of patients, although contraindicated combinations were found to be relatively rare. The comedications most commonly associated with ARV DDIs in this cohort were anti-infectives. Pharmacokinetic mechanisms involving drug metabolism accounted for the majority of interactions. The patients at greatest risk of DDIs were those taking two or more comedications, taking a PI-containing regimen, taking an anti-infective (antibiotic, antifungal, anthelmintic or antimalarial) or WHO clinical stage 3–4. By prioritizing patients with two or more of these risk factors for DDI screening, up to 90% of the patients with DDIs would be identified via screening of <50% of the cohort. The available resources would determine the choice of risk score model, depending on the feasibility of prioritizing ∼28% of patients for screening to detect 75% of patients with DDIs (Model 2) or prioritizing ∼50% of patients to detect almost 90% of DDIs (Model 1). The method used would also depend on the simplicity of the scoring system in the clinical context, e.g. whether the healthcare worker screening patients has reliable information on current medication and/or WHO clinical stage. WHO stage may represent a simple measure that could be identified by a trained healthcare worker or lay person, in order to flag up high-risk patients for DDI screening (Model 3). In this setting, it may be possible that anti-infectives or other comedications are supplied to patients from outside the clinic and these medicines may not be recorded in clinical notes. If high-risk patients (WHO stage 3–4, PI regimens and/or at least two comedications) are prioritized, they can undergo DDI screening, which includes a full medication history.

Potential DDIs involving ARVs occur at similar rates in resource-limited settings and developed countries. Drug combinations that most frequently cause DDIs, however, differ between settings, e.g. CNS and cardiovascular drugs in the UK^3,7^ and anti-infectives in Kenya^10^ and Uganda. There are some differences in risk factors, e.g. age confers a higher risk of DDIs in European studies,^6^ whereas no significant association was found in this cohort. Higher number of comedications and PI-based regimens are predictors for DDI risk in this cohort and European settings.^3,7^ A study in Argentina found that DDIs affected ∼30% of patients and most frequently occurred with anti-infectives, CNS drugs and cardiovascular drugs. Age was not associated with increased risk of DDIs, nor was a PI-containing regimen. Only at least two comedications and taking a CNS drug were significantly associated with higher risk of DDIs.^8^ As prescribing patterns and medication use in sub-Saharan Africa change, to include more widespread prescribing of medicines used in chronic conditions and conditions associated with advanced age, e.g. CNS and cardiovascular agents, it is likely that the type of and risk factors for DDIs with ARVs will change, potentially with some similarities to the data observed in Argentina and Europe. However, these data suggest the overall prevalence of potential DDIs is unlikely to decrease, in the absence of targeted interventions.

Healthcare provision in many resource-limited settings involves independent silos of healthcare delivery, e.g. vertical (unintegrated) HIV services, TB treatment and programmes for treatment of other infectious conditions. This undoubtedly complicates the communication process and increases the risk of unrecognized DDIs. Taking and recording full drug histories from patients is therefore important in all healthcare settings. As patients were not approached to give a full medication history, and due to the often unintegrated (vertical) nature of treatment programmes in this setting and potential for widespread use of over-the-counter (OTC) and traditional herbal medicines,^14^ these results represent a conservative estimate of the true prevalence of DDI in this setting.

In addition, the centre at which the study took place is a large, relatively well-resourced facility in Kampala, with electronic patient records and partial electronic prescribing. The results therefore may not be representative of all centres that provide HIV services in Uganda, or the rest of sub-Saharan Africa, where systems and available regimens may differ. Again, this study is likely to represent a conservative estimate of the true prevalence of DDIs in ARV programmes in sub-Saharan Africa. Future work will use an in-depth approach and evaluate the prevalence of DDIs with full medication lists for each patient, including OTC and herbal or traditional medicines, and include peripheral, more representative centres that provide HIV services.

Using these data, health system interventions will be developed including prescribing alerts and screening for DDIs in clinic. Such interventions will be assessed for utility and reduction in patient harm. For example, in settings where not all patients are routinely checked for DDIs, due to time, staff or resource constraints, or where internet access for online DDI resources is limited, a screening tool can be used to identify patients at high risk. Trained lay workers, or other healthcare workers involved in patient care, could be trained to use the screening tool and flag-up high-risk patients prior to outpatient appointments, so that these patients can be prioritized for a DDI check. It may be possible to train healthcare workers such as pharmacy technicians to check for DDIs using a printed chart or, where available, an online database. Another option would be to put up posters in clinics, displaying the factors that confer higher risk of DDIs (i.e. WHO stage 3–4, two or more comedications, use of anti-infectives and a PI-containing ARV regimen) and suggesting that patients with two or more of these risk factors be screened for DDIs.

Potential DDIs involving ARVs occur at similar rates in resource-limited settings and developed countries, although the comedications most commonly associated and risk factors differ. Development of tools, including databases that incorporate drugs in common use throughout sub-Saharan Africa, is essential for recognition of DDIs. Understanding risk factors for DDIs in specific settings can allow patients with known risk factors to be prioritized for DDI screening in settings with low resources. An understanding of the key DDIs of clinical relevance in specific settings can allow introduction of interventions such as alerts on electronic prescribing systems and standard local guidance for interaction management in settings with protocol-driven treatment programmes.

## Funding

This study was supported by internal funding.

K. S. is supported by a Medical Research Council Population Health Science Fellowship (MR/L012413/1). M. L. is supported by a European and Developing Countries Clinical Trials Partnership Senior Fellowship (TA.2011.40200.047).

## Transparency declarations

K. S. has received speaker fees from ViiV and travel bursaries from Merck, ViiV and Gilead. M. S. has received research grants from ViiV and Janssen. M. L. has received research grants from Janssen and ViiV. R. P.-R. has received consultancy fees from Janssen Global Public Health. D. B. and S. K. have received honoraria or research grants from Boehringer Ingelheim, Bristol-Myers Squibb, Gilead Sciences, Janssen, Merck, Roche and ViiV. All other authors: none to declare.

## Supplementary data

Figure S1 is available as Supplementary data at *JAC* Online (http://jac.oxfordjournals.org/).

Supplementary Data
