# Supplementary material for: Prevalence and type of drug–drug interactions involving ART in patients attending a specialist HIV outpatient clinic in Kampala, Uganda
Source: J Antimicrob Chemother. 2015 Aug 18;70(12):3317–22. doi: 10.1093/jac/dkv259 (PMC4652684; doi:10.1093/jac/dkv259)
Supplement: Supplementary Data [file supp_70_12_3317__index.html]

Prevalence and type of drug–drug interactions involving ART in patients attending a specialist HIV outpatient clinic in Kampala, Uganda — Prevalence and type of drug–drug interactions involving ART in patients attending a specialist HIV outpatient clinic in Kampala, Uganda — Supplementary Data 

# Prevalence and type of drug–drug interactions involving ART in patients attending a specialist HIV outpatient clinic in Kampala, Uganda

## Supplementary Data

Supplementary Data

- Supplementary Data - Docx file
