# Supplementary material for: Prevalence and type of drug–drug interactions involving ART in patients attending a specialist HIV outpatient clinic in Kampala, Uganda
Source: J Antimicrob Chemother. 2015 Aug 18;70(12):3317–22. doi: 10.1093/jac/dkv259 (PMC4652684; doi:10.1093/jac/dkv259)
Supplement: Supplementary Data [file supp_dkv259_dkv259supp.docx]

**Supplementary data**

**Figure S1.** Co-medication usage and prevalence of DDIs between ARVs and different classes of co-medication. NB: 1848 patients take PCP prophylaxis; *x*-axis capped at 500 patients.
